# Prevalence of and relevant factors for depression and anxiety symptoms among pregnant women on the eastern seaboard of China in the post-COVID-19 era: a cross-sectional study

**DOI:** 10.1186/s12888-023-05059-2

**Published:** 2023-08-07

**Authors:** Haidong Yang, Yangyang Pan, Wanming Chen, Xu Yang, Bin Liu, Nian Yuan, Xiaobin Zhang

**Affiliations:** 1 Department of Psychiatry, The Fourth People’s Hospital of Lianyungang, The Affiliated KangDa College of Nanjing Medical University, Lianyungang, 222003 P.R. China; 2 Department of Psychiatry, Beijing Hui Long Guan Hospital, Peking University Hui Long Guan Clinical Medical School, Beijing, 100096 P.R. China; 3 Science and Education Section, The Fourth People’s Hospital of Lianyungang, The Affiliated KangDa College of Nanjing Medical University, Lianyungang, 222003 P.R. China; 4 Institute of Mental Health, Suzhou Psychiatric Hospital, The Affiliated Guangji Hospital of Soochow University, Suzhou, 215137 P.R. China

**Keywords:** Antenatal depression, Antenatal anxiety, Prevalence, Risk factors, Pregnant women, Post-COVID-19 era

## Abstract

**Background:**

Antenatal depression and anxiety symptoms may have negative consequences for both mothers and offspring, and upward trends in the prevalence of these symptoms were especially apparent during the COVID-19 epidemic. The purpose of this study was to evaluate the prevalence of and relevant factors influencing depressive and anxiety symptoms in Chinese pregnant women in the post-COVID-19 era.

**Methods:**

We conducted an online survey of 1,963 pregnant women in Jiangsu Province, using a cross-sectional design, and collected their general demographic data. The nine-item Patient Health Questionnaire 9 (PHQ-9) was used to evaluate depression symptoms, and the seven-item Generalized Anxiety Disorder 7 (GAD-7) was used to measure anxiety symptoms.

**Results:**

The prevalence of reported antenatal depressive symptoms, anxiety symptoms, and depression combined with anxiety symptoms was 25.2%, 27.9%, and 18.6%, respectively. Of the respondents, the prevalence of moderate to severe depression, and anxiety was 7.9% and 7.7%, respectively. Binary logistic regression analysis demonstrated that age, low level of education, rural area, unemployment, pregnancy complications, poor marital relationship, and fair household income were positively association with both depressive and anxiety symptoms (all *P* < 0.05). The proportion of women reporting anxiety symptoms in the third trimester was 1.91-fold higher than in first trimester. Parity was a relevant factor for depression and anxiety symptoms (all *P* < 0.05).

**Conclusions:**

In the post-COVID-19 era, the prevalence of depression and anxiety symptoms in pregnant women was higher than expected, and it is vital to establish hospital, community, and family psychological health screening systems based on relevant factors and enhance early preventive measures.

## Introduction

Depression and anxiety have now been recognized as complicated and widespread mental disorders, the etiologies of which probably encompass heterogeneous genetic, environmental, and biological factors, and whose exact pathophysiological mechanisms remain obscure, resulting in a major illness burden [1–4]. According to the Global Burden of Disease 2019 [5], depression and anxiety disorders were the second and eighth leading causes of years lived with disability, respectively, among the top 25 causes across all age categories. The age-standardized disability-adjusted life-years rate of mental illnesses is higher for females than for males and some specific populations such as pregnant women appear to be more vulnerable to the anxiety and depressive symptoms that accompany role, psychological, and physical transitions [6].

Antenatal depression and anxiety symptoms can have adverse consequences for both the pregnant woman and her child. These include postpartum depression, preterm delivery, low birth weight, malnutrition, and changes to social-emotional and behavioral functioning [7–9]. With 2.73% of pregnant women reporting suicidal thoughts, prenatal depression has emerged as a serious public health concern [10]. Antenatal depression and anxiety symptoms have become an imperative to be addressed across countries and regions [11, 12].

Numerous studies already available have reported many factors involved in antenatal depression and anxiety. For example, a systematic review reported that in low- and middle-income countries, physical, psychological, or sexual abuse in early life or in adulthood, low maternal education level, lower socioeconomic situation, and lack of social support were risk factors for depression in pregnant women [8]. A cross-sectional study reported that poor sleep quality was related to antepartum suicidal ideation among pregnant women in the United States of America (USA) [13]; another study from Saudi Arabia reported that absence of family or partner support, history of cesarean section, parity, and abortion were possible risk factors [14]. The prevalence of depression and anxiety symptoms varies by trimester, with higher incidences in the first and third trimesters than in the second trimester [15, 16]. In addition, biological factors such as cytokines [17] and levels of dopamine, serotonin, cortisol and norepinephrine [18] may also be responsible for symptoms of depression or anxiety. Resilience, on the other hand, has been shown in another study to be a possible protective factor in pregnancy [19].

Notably, the COVID-19 outbreak also contributed to the dire status of mental healthcare in most countries, with a marked increase in prenatal depression and anxiety symptoms [20–22]. During the COVID-19 pandemic, a rapid review and meta-analysis reported the pooled prevalence of depression and anxiety in pregnant women as 25.6% and 30.5%, respectively [23]. Prevalence varied between countries, with depressive symptoms among 37% of pregnant women in Canada, 25% in Belgium, 35.4% in Turkey, 19.5% in Sri Lanka, and 11–29% in China. Anxiety symptoms were reported in 38% of pregnant women in Italy, 56% in Canada, 14% in Pakistan, 53% in Greece, 17.5% in Sri Lanka, 3.8% in Iran, and 0.3–29% in China during the COVID-19 period [24]. The COVID-19 epidemic obviously impacted negatively on the mental health of pregnant women, who may experience complex stress and psychological problems during pregnancy.

Prenatal depression and anxiety have been widely discussed and their prevalence and influencing factors need investigating with the aims of developing relevant prevention strategies and reducing negative health effects. Reviews have been conducted previously to assess the prevalence of and risk factors for depression and anxiety in pregnant women. For example, an umbrella review including 306 studies reported that the prevalence of antenatal depression was in the range of 15–65% [25], and a systematic review and meta-analysis that included 700 full-texts reported a pooled prevalence of 29.2% for self-reported anxiety symptoms [26]. Nevertheless, results have been inconsistent, regardless of the study investigation method, geographic location, and cultural background.

In the post-COVID-19 era, COVID-19 is essentially under control, but the epidemic may ebb and flow at any time, and small outbreaks may continue for longer periods of time and have profound consequences on various aspects of people’s lives [27]. Based on previous studies have examined the prevalence of prenatal depression and anxiety symptoms at different stages and in different countries [13, 14, 19, 24], our understanding of the prevalence, risk factors, and symptoms of depression and anxiety among pregnant women in China during the post-COVID-19 era remains limited. Therefore, the purpose of this study was to investigate the prevalence and risk factors of antenatal depressive symptoms and anxiety symptoms among pregnant women in the post-COVID-19 era, as well as to analyze the relationship between them. Additionally, providing pertinent coping strategies is also important. For instance, we propose intervention strategies based on risk factors in the post-COVID-19 era rather than the previous pandemic stages, to promote health care and establish a mental health system linking families, hospitals and communities, to enhance the mental health of pregnant women. Although there have been prior studies conducted [28–30], it is important to further explore the impact of the post-COVID-19 era on the mental health of pregnant women in order to expand our knowledge. We hypothesized that the prevalence of prenatal depression and anxiety symptoms may be higher, and that various demographic and socioeconomic factors including region, employment status, gestational age, complications, marital relationship, and annual household income may contribute to the risk factors associated with these symptoms. To our knowledge, to date, few studies have investigated the prevalence and correlates of depression and anxiety symptoms among pregnant women in the post-COVID-19 era [31].

## Methods

### Procedures and subjects

A cross-sectional survey was conducted in Lianyungang, Jiangsu Province, China, between August 2021 and February 2022. An anonymous online questionnaire was used on the Wenjuanxing management platform (https://www.wjx.cn/app/survey.aspx) to collect data. The link to the survey was distributed through primary health care institutions located in the six administrative districts of Lianyungang, and the respondents were required to be pregnant women, as determined by the date of the last menstrual period and B ultrasound results. Respondent involvement was voluntary, and the respondents were instructed in detail about the objectives, content, procedures, and confidentiality of the data prior to taking part in the online survey, finally signing informed consent. Respondents could withdraw at any time if they did not wish to continue completing the survey. The study was approved by the Ethics Committee of the Fourth People’s Hospital of Lianyungang City.

### Measure

A sociodemographic data questionnaire was designed by the researchers, which collected information on age, education (junior high school or less/senior high school/college or more), residential location (urban/rural area), post-pregnancy working status (employed/unemployed), gestation age (first, second, third trimester), parity (0, ≥ 1), pregnancy complications (yes/no), and marital relationship (poor/moderate/satisfying). Annual household income status corresponding to fair, good, and better was below ¥100,000, ¥100,000 to ¥200,000, and above ¥200, 000, respectively.

Antenatal depression symptoms were assessed using the Patient Health Questionnaire 9 (PHQ-9), which is a nine-item self-assessment tool based on the diagnostic criteria for depressive disorder of the DSM-IV. The PHQ-9 is considered to have proven adaptability, reliability, and validity in the Chinese population [32, 33]. The PHQ-9 consists of nine items evaluating the experiences of respondents suffering from depressive symptoms in the past 2 weeks. It is scored using a four-point Likert scale for each item: from 0 (not at all), 1 (several days), 2 (more than half the days), to 3 (nearly every day). The total score ranges from 0 to 27, with severity ranging from 0 to 4 as minimal, 5–9 as mild, 10–14 as moderate, 15–19 as moderately severe, and 20–27 as severe in accordance with the recommended criteria of Kroenke et al. [34]. Participating individuals were considered to have depressive symptoms if they had a score of 5 or higher [35, 36]. Cronbach’s alpha value of the PHQ-9 was 0.879.

We used the seven-item Generalized Anxiety Disorder Scale 7 (GAD-7) to assess antenatal anxiety symptoms, which has been verified as a validated anxiety self-rating scale among pregnant Chinese women [37]. The questionnaire was used to screen the severity of anxiety symptoms experienced in the past 2 weeks on a four-point Likert scale, with each item ranging from 0 to 3, with a score of 0 for “not at all”, a score of 1 for “several days”, a score of 2 for “more than half the days”, and a score of 3 for “nearly every day”. The GAD-7 has seven items scored for a maximum total score of 21 points, based on Spitzer et al. [38], with scores ranging from 0 to 4 classified as minimal, 5–9 as mild, 10–14 as moderate, and 15–21 as severe anxiety symptoms. A cut-off of 5 points or higher has been suggested to indicate possible anxiety symptoms [39]. Cronbach’s alpha coefficient of the GAD-7 was 0.941. In accordance with the PHQ-9 and GAD-7 severity cut-off scores, pregnant women with a PHQ-9 total score of at least 5 and a GAD-7 total score of at least 5 were considered to have comorbid depression and anxiety [35, 36, 39].

### Statistical analysis

The Statistical Product and Service Solutions version 19.0 (SPSS 19.0) was used for the dataset analyses. Continuous variables were expressed as mean and standard deviation (SD), to which Student’s *t*-test or independent samples t-test was applied, and categorical variables were expressed as percentages with chi-square test used. Univariate analysis was performed to validate possible risk factors for anxiety and depressive symptoms. We identified the presence of depressive symptoms or the presence of anxiety symptoms as dichotomous variables and used logistic regression analysis to determine the relationship between depressive symptoms and other variables to evaluate predictors. P values below 0.05 were considered to be statistically significant (two-tailed test).

## Results

### Sociodemographic characteristic of participants

A total of 2,011 pregnant women responded to the survey; of these, 35 (1.74%) were excluded for not completing necessary items such as age, trimester, and questionnaire content, and 13 (0.65%) pregnant women who had a history of mental disorder prior to the survey were excluded. Ultimately, the responses of 1,963 pregnant women were included in the analysis. All 1,963 included women were aged between 18 and 42 years (29.81 ± 4.51). The average duration of education for respondents was 13.36 ± 2.47 years, with the proportions completing junior high school or below, senior high school, and college or above 10.6% (n = 208), 47.1% (n = 924), and 42.3% (n = 831), respectively. The percentage of pregnant women living in urban areas was 65.8% (n = 1291). Of all participants, 68.2% (n = 1338) were still working during pregnancy. A total of 37.2% (n = 730) of respondents were in their first trimester, 28.1% (n = 552) were in the second trimester, and 34.7% (n = 681) were in the third trimester. Primiparous women comprised 51.1% (n = 1001) and 48.9% (n = 960) were multiparous, and 6.1% (n = 119) had comorbid somatic diseases. The proportions of women reporting their marital relationship as satisfying, moderate, and poor were 51.0% (n = 1001), 29.3% (n = 575), and 19.7% (n = 387), respectively. The proportions of households reporting high, good, and fair economic status were 14.2% (n = 278), 62.6% (n = 1229), and 23.2 (n = 456), respectively, as shown in Table 1.

**Table 1:** Sociodemographic and clinical characteristics of pregnant women (n = 1963)

	Depression symptoms			Anxiety symptoms		
	With (n = 1468)	Without (n = 495)	P	With (n = 1416)	Without (n = 547)	P
Age (years), mean (SD)	32.6 (3.7)	28.9 (4.4)	< 0.001^a^	31.3 (4.4)	29.2 (4.4)	< 0.001^a^
Education (years), mean (SD)	14.3 (2.6)	13.1 (2.3)	< 0.001^a^	13.7 (2.6)	13.2 (2.4)	< 0.001^a^
Educational level, n (%)			< 0.001^b^			< 0.001^b^
Junior high school or below	63(30.3)	145(69.7)		63(30.3)	145(69.7)	
Senior high school	105(11.4)	819(88.6)		202(21.9)	722(78.1)	
College or above	327(39.4)	504(60.6)		282(33.9)	549(66.1)	
Region, n (%)			0.032^b^			< 0.001^b^
Urban	306(23.7)	985(76.3)		326(25.3)	965(74.7)	
Rural	189(28.1)	483(71.9)		221(32.9)	451(67.1)	
Working status, n (%)			< 0.001^b^			< 0.001^b^
Employment	306(22.9)	1032(77.1)		337(25.2)	1001(74.8)	
Unemployment	189(30.2)	436(69.8)		210(33.6)	415(66.4)	
Gestation age, n (%)			0.294^b^			< 0.001^b^
First trimester	175(24.0)	555(76.0)		133(18.2)	597(81.8)	
Second trimester	134(24.3)	418(75.7)		162(29.3)	390(70.7)	
Third trimester	186(27.3)	495(72.7)		252(37.0)	429(63.0)	
Parity, n (%)			0.098^b^			0.725^b^
0	237(23.6)	766(76.4)		276(27.5)	727(72.5)	
≥ 1	258(26.9)	702(73.1)		271(28.2)	689(71.8)	
Complications, n (%)			< 0.001^b^			< 0.001^b^
Yes	82(68.9)	37(31.1)		86(72.3)	33(27.7)	
No	413(22.4)	1431(77.6)		461(25.0)	1383(75.0)	
Marital relationship, n (%)			< 0.001^b^			< 0.001^b^
Satisfying	167(16.7)	834(83.3)		141(14.1)	860(85.9)	
Moderate	77(13.4)	498(86.6)		161(28.0)	414(72.0)	
Poor	251(64.9)	136(35.1)		245(63.3)	142(36.7)	
Annual household income, n (%)			< 0.001^b^			< 0.001^b^
Fair	125(45.0)	153(55.0)		138(49.6)	140(50.4)	
Good	287(23.4)	942(76.6)		308(25.1)	921(74.9)	
Better	83(18.2)	373(81.8)		101(22.1)	355(77.9)	

a Independent samples t-test. b χ^2^ test. SD standard deviation

### Prevalence of depressive and anxiety symptoms among pregnant women

As shown in Table 2, the overall prevalence of reported antenatal depression symptoms, antenatal anxiety symptoms, and antenatal comorbid depressive and anxiety symptoms was 25.2%, 27.9%, and 18.6%, respectively. A significant difference was found in the prevalence of depression and anxiety symptoms among pregnant women by educational level, which were higher in those with education levels of junior high school or below and college or above than senior high school (χ^2^ = 184.894, *P* < 0.001; χ^2^ = 32.408, *P* < 0.001, respectively). Compared with women in urban areas, pregnant women in rural areas had more symptoms of depression and anxiety (χ^2^ = 4.584, *P* = 0.032; χ^2^ = 12.817, *P* < 0.001, respectively). In addition, unemployed women had greater levels of depression (χ^2^ = 12.271; *P* < 0.001) and anxiety (χ^2^ = 15.001; *P* < 0.001), as did women with physical illness (depression: χ^2^ = 128.233, anxiety: χ^2^ = 124.259; *P* < 0.001). Pregnant women with satisfying marital relationships reported fewer depression symptoms (χ^2^ = 403.780; *P* < 0.001) and fewer anxiety symptoms (χ^2^ = 336.408; *P* < 0.001), as did those with higher annual household income (depression: χ^2^ = 71.652, anxiety: χ^2^ = 77.798, both *P* < 0.001). No significant difference was observed in the prevalence of depressive symptoms between gestational stages (*P* > 0.05); however, the prevalence of anxiety symptoms increased significantly in the third trimester compared with the first and second trimesters (χ^2^ = 62.693, *P* < 0.001). No significant difference was found in the prevalence of depressive and anxiety symptoms according to parity (all *P* > 0.05).

The percentage of respondents with mild, moderate, moderate to severe, and severe depressive symptom severity was 17.3%, 5.5%, 1.6%, and 0.8%, respectively. Meanwhile, the proportions of respondents with anxiety symptoms of severity levels of mild, moderate, and severe were 20.2%, 5.7%, and 2.0%, respectively. Furthermore, the percentages of women with depressive symptoms, anxiety symptoms, and depression combined with anxiety symptoms ranging from moderate to severe were 7.9% and 7.7%, respectively, as shown in Table 2, and the average scores for different severity levels of depressive symptoms and anxiety symptoms are also presented.

**Table 2:** Percentage of pregnancies with various depression and anxiety symptom severity levels

	Depressive symptoms		Anxiety symptoms	
	N (%)	Mean (SD)	N (%)	Mean (SD)
Minimal	1468 (74.8)	1.95 (1.62)	1416 (72.1)	0.83 (1.29)
Mild	339 (17.3)	6.35 (1.34)	396 (20.2)	6.53 (1.10)
Moderate	108 (5.5)	11.93 (1.39)	112 (5.7)	11.92 (1.49)
Moderately severe	32 (1.6)	16.0 (1.32)	-	-
Severe	16 (0.8)	22.31 (1.82)	39 (2.0)	18.79 (2.33)
Moderate to severe	156 (7.9)	13.83 (3.59)	151 (7.7)	13.71 (3.47)
Mild to severe	495 (25.2)	8.71 (4.17)	547 (27.9)	8.51 (3.81)

SD standard deviation

### Analyses of associations between the prevalence of depression and anxiety symptoms among pregnant women

Univariate analyses demonstrated no statistically significant difference in the prevalence of depressive symptoms for trimester and parity (all *P* > 0.05), and no significant difference in the prevalence of anxiety symptoms for parity (*P* > 0.05); therefore, trimester was not included in the binary logistic analysis of depression symptoms. However, because a previous study has suggested that parity may also influence depression or anxiety symptoms [21], we included parity in the logistic regression analysis to reduce bias in the results. Also, the prevalence of reported depression and anxiety symptoms showed significant differences among ages (P < 0.001); accordingly, age was included in the regression equation for depression and anxiety symptoms.

As shown in Table 3, regarding antenatal depressive symptoms, binary logistic regression showed that age and living in a rural region were positively associated with depressive symptoms (B = 0.108, *P* < 0.001, odds ratio (OR) = 1.114, 95% confidence interval (CI): 1.074–1.156; B = 0.953, *P* < 0.001, OR = 2.592, 95% CI: 1.905–3.526, respectively). Similar results were revealed for being unemployed and for the presence of complications during pregnancy (B = 0.528, *P* < 0.001, OR = 1.695, 95% CI: 1.275–2.254; B = 2.007, *P* < 0.001, OR = 7.442, 95% CI: 4.430–12.50, respectively). A positive association was observed between multiparity, rather than primiparity, and depressive symptoms (B = 0.874, *P* < 0.001, OR = 2.398, 95% CI: 7.765–3.257). Compared with junior high school education or below, education at senior high school was negatively related to depressive symptoms (B = -1.229, *P* < 0.001, OR = 0.292, 95% CI: 0.180–0.476). Poor marital relationship showed a significantly positive correlation with depression compared with satisfying marital relationship (B = 2.535, *P* < 0.001, OR = 12.611, 95% CI: 8.803–18.064). Good and high annual household income were negatively associated with depressive symptoms compared with fair annual household income (B = -2.049, *P* < 0.001, OR = 0.129, 95% CI: 0.082–0.204; B = -2.078, *P* < 0.001, OR = 0.125, 95% CI: 0.076–0.206, respectively).

In terms of antenatal anxiety symptoms, binary logistic regression showed that age (B = 0.078, *P* < 0.001, OR = 1.081, 95% CI: 1.047–1.117), rural region (B = 0.644, *P* < 0.001, OR = 1.904, 95% CI: 1.457–2.489), unemployed working status (B = 0.441, *P* < 0.001, OR = 1.554, 95% CI: 1.215–1.989), and pregnancy complications (B = 2.158, *P* < 0.001, OR = 8.650, 95% CI: 5.340–14.012) were positively associated with anxiety symptoms. Compared with junior high school level or below, education at senior high school and college level or above were negatively related to anxiety symptoms (B = -0.654, *P* = 0.002, OR = 0.525, 95% CI: 0.348–0.791; B = -0.488, *P* = 0.026, OR = 0.614, 95% CI: 0.399–0.944, respectively). Poor marital relationship was positively associated with anxiety symptoms compared with moderate and satisfying martial relationship (B = 0.811, *P* < 0.001, OR = 2.250, 95% CI: 1.622–3.119; B = 2.072, *P* < 0.001, OR = 7.940, 95% CI: 5.827–10.821, respectively). Also, in women with good and high annual household income there was a negative association with anxiety symptoms, compared with those with fair annual household income (B = -1.252, *P* < 0.001, OR = 0.286, 95% CI: 0.196–0.417; B = -1.224, *P* < 0.001, OR = 0.294, 95% CI: 0.195–0.445, respectively). Moreover, the third trimester was shown to be positively related to anxiety symptoms compared with the first trimester (B = 0.647, *P* < 0.001, OR = 1.910, 95% CI: 1.370–2.661); a positive association was observed between multiparity, rather than primiparity, and anxiety symptoms (B = 0.512, P < 0.001, OR = 1.669, 95% CI: 1.276–2.183).

**Table 3:** Risk factors related to the prevalence of depression and anxiety symptoms

	Depressive symptoms			Anxiety symptoms		
	OR	95%CI	P	OR	95%CI	P
Age	1.114	1.074–1.156	< 0.001	1.081	1.047–1.117	< 0.001
Educational level						
Junior high school or below	1		< 0.001	1		0.009
Senior high school	0.292	0.180–0.476	< 0.001	0.525	0.348–0.791	0.002
College or above	2.101	1.321–3.340	0.002	0.614	0.399–0.944	0.026
Region						
Urban	1			1		
Rural	2.592	1.905–3.526	< 0.001	1.904	1.457–2.489	< 0.001
Working status						
Employment	1			1		
Unemployment	1.695	1.275–2.254	< 0.001	1.554	1.215–1.989	< 0.001
Gestation age						
First trimester	1		< 0.001	1		< 0.001
s trimester	0.662	0.434–1.008	0.055	1.398	0.969–2.017	0.073
Third trimester	0.369	0.257–0.530	< 0.001	1.910	1.370–2.661	< 0.001
Parity						
0	1			1		
≥ 1	2.398	1.765–3.257	< 0.001	1.669	1.276–2.183	< 0.001
Complications						
No	1			1		
Yes	7.442	4.430–12.500	< 0.001	8.650	5.340–14.012	< 0.001
Marital relationship						
Satisfying	1		< 0.001	1		< 0.001
Moderate	0.842	0.570–1.244	0.387	2.250	1.622–3.119	< 0.001
Poor	12.611	8.830–18.064	< 0.001	7.940	5.827–10.821	< 0.001
Annual household income						
Fair	1		< 0.001	1		< 0.001
Good	0.129	0.082– 0.204	< 0.001	0.286	0.196–0.417	< 0.001
Better	0.125	0.076–0.206	< 0.001	0.294	0.195–0.445	< 0.001

## Discussion

To our knowledge, our study was the first cross-sectional investigation of the prevalence of and risk factors for antenatal depression and anxiety symptoms among pregnant women on the eastern seaboard of China in the post-COVID-19 era. The study showed that in the post-pandemic era of COVID-19, the prevalence of antenatal depressive and anxiety symptoms was 25.2% and 27.9%, respectively, and 18.6% of pregnant women had a combination of depression and anxiety symptoms. Many factors were found to be potentially related to antenatal depression and anxiety symptoms, including age, education, rural area, work during pregnancy, comorbid somatic illnesses, poor marital relationship, and household economic status. Additionally, gestation age had a noticeable influence on antenatal anxiety symptoms.

Surveys on the prevalence of depression and anxiety in pregnant women have been ongoing, but results have been inconsistent. For example, during the COVID-19 epidemic, a systematic review and meta-analysis that included 10 countries reported the overall prevalence of depression and anxiety to be 25% and 42%, respectively [40]. Several cross-sectional studies have reported a prevalence of depression and anxiety symptoms among pregnant women of 37% and 57%, respectively, in Canada and 18.1% and 45.9%, respectively [21, 41]. The inconsistent results may be attributed to sample size, ethnicity, geography, cultural background, and the survey tool used. For example, in these studies, the sample sizes ranged from 205 to 1987 or more; the regions included the USA, Canada, Iran, and China, each of which have populations with different ethnic and cultural backgrounds and different attitudes toward mental illness [42]; the survey instruments varied in their evaluation criteria; and the research methods included cross-sectional studies, and systematic reviews and meta-analyses. Regardless of these differences, a commonality is that the mental health of pregnant women warrants concern. Our study showed that the proportions of women with antenatal depression and antenatal anxiety symptoms were 25.2% and 27.9% in the post-epidemic era, and the reported results, compared with those during COVID-19, indicated a prevalence of prenatal anxiety and depression that was moderate yet still higher than that from the pre-COVID-19 era [43]. This suggested that the COVID-19 epidemic may have had long-term consequences on the psychological health of pregnant women, which is a cause for concern.

Previous studies have shown that depression and anxiety symptoms may be highly comorbid due to the presence of common contributory factors [44, 45]. In the current study, we found that the prevalence of comorbid depression and anxiety symptoms among pregnant women was 18.6%. A multicultural cross-sectional study conducted before the outbreak of COVID-19 reported a 26.9% prevalence of comorbid depression and anxiety in the early stages of pregnancy, ranging from 47.6% in the Turkish cohort to 9.5% in Spanish pregnant women [46]. During the COVID-19 pandemic, the prevalence of comorbid anxiety and depression was 5.8% among pregnant individuals in China [47] and 3.95% in Nigeria [48]. These comparisons, despite the differing results, suggested that the COVID-19 pandemic contributed to an increase in depression and anxiety symptoms in pregnant women. Although our results showed a decrease in reported anxiety and depression symptoms, this decrease was slight, and it was possible that the multifaceted effects attributed to the COVID-19 epidemic persisted into the post-epidemic era. A multinational cohort study has demonstrated that neonates born to pregnant women infected with COVID-19 have a significantly higher severe morbidity index, and a severe perinatal morbidity and mortality index [49]. Studies have indicated that the recession and social isolation resulting from COVID-19 may increase the incidence of depression, anxiety, sleep disorders, substance use, or suicidal behavior in individuals [50, 51]. These results warrant further investigation as a starting point in developing new strategies for public health.

Our findings demonstrated that the proportions of pregnant women reporting moderate to severe depression and anxiety were 7.9% and 7.7%, respectively, in the post-COVID-19 era. Based on the consistent use of the PHQ-9 and GAD-7 screening scales, we observed a decrease in prenatal depression and anxiety, compared with the COVID-19 epidemic period. These findings were not entirely consistent with other studies. For example, in China, during the COVID-19 epidemic, the prevalence of reported moderate to severe depressive symptoms and of anxiety symptoms was 9.1% and 9.8%, respectively [52, 53]. Nevertheless, caution should be applied when making direct comparisons, as the heterogeneity of results may be related to sociodemographic profiles, sampling methods, and different phases of the epidemic. It is worth noting, however, that during the COVID-19 pandemic, the increased uncertainty exerted an adverse psychological impact on pregnant women, contributing to an elevated prevalence of depression and anxiety symptoms [54]. Importantly, regardless of the pandemic stage, it is necessary to ascertain early in pregnancy the likelihood of mental health concerns in women so that psychological services can be provided to manage short- and long-term effects.

A previous study indicated that stressful events may trigger negative thought patterns and self-blame, and enhance the likelihood of depressive and anxiety symptoms developing in pregnant women [55]. Many co-existing factors may also impact on the psychological state of pregnant women, such as age, years of education, employment, area of residence, trimester, parity, pregnancy complications, and access to information [56]. In addition, better social support and higher levels of household income have been found to be related to fewer depressive and anxiety symptoms [40]. In the present study we found that age, educational level, rural area, joblessness, pregnancy with physical illness, poor marital relationship, and general family economic situation were risk factors for depression and anxiety symptoms in pregnancy, which was consistent with previous studies [57–59]. Variations in sociodemographic factors affect the psychological transformation of pregnant women as individuals adopting new responsibilities and roles during pregnancy, and positive sociodemographic factors may play a role in social support and reduce the adverse consequences of stressful events on the psychological well-being of pregnant women [60]. Therefore, considering the consequences of these psychosocial factors, it is necessary to take measures to prevent and manage prenatal anxiety and depression in response to psychosocial factors, and providing additional social support, cognitive-behavioral therapy, and interpersonal therapy to pregnant women may decrease depression and anxiety rates.

Interestingly, our findings indicated that third trimester gestation may be associated with a greater likelihood of prenatal anxiety symptoms. One reason for this result may be related to concerns about infant health and delivery in the context of the COVID-19 epidemic, and another may be associated with a lack of intervention for anxiety symptoms in early pregnancy, which may progressively reinforce anxiety symptoms [61, 62]. Another reason is that third trimester gestational age is associated with a significant increase in COVID-19-related stressors, and experiencing longer COVID-19, with longer duration of stress, may cause more anxiety [63, 64]. Previous studies have shown that the third trimester is the most physically burdensome period for pregnant women, and that pain during this period triggers depression, anxiety symptoms, and even suicidal ideation [65, 66]. The sociodemographic characteristics of pregnant women, a high-risk group for depression and anxiety, have been partially presented, but these may not provide the whole picture. Mounting evidence indicates that the hypothalamic-pituitary-adrenal axis, autonomic nervous system, neurotransmitters, cytokines and chemokines may be involved in depression and anxiety during pregnancy, and further research is required [17, 67, 68] .

Our findings have clinical and policy implications. Depression and anxiety symptoms in pregnant women have become a major public health problem, and the prevalence remains high in the post-COVID-19 era as a result of the epidemic and other factors, creating a large burden to families and individuals and disrupting the psychosomatic health of pregnant women. First, it is important to provide policymakers with credible data from the evaluation of high-risk groups in pregnancy, to enable the recommendation of strategies for management and response. Second, the entire antenatal period is monitored, and mental health screening can be included in health checkups, continuing after delivery. Pregnant women with initial symptoms can be guided to proactively seek professional diagnosis. Third, intervention strategies such as psychotherapy should be implemented as soon as possible for pregnant women with moderate to severe depression and anxiety symptoms, to enable the development of psychological resilience. Moreover, it is vital to establish an accessible and comprehensive mental health screening system that includes hospitals, communities, and families, and to step up education on early prevention.

The present study had several limitations. First, the use of self-reported scales might have resulted in recall bias in the results. Second, the study was cross-sectional and may not demonstrate the causality of risk factors in relation to depression and anxiety symptoms in pregnancy. Long-term longitudinal studies to explore this are warranted. Third, we did not explore other risk factors associated with depression and anxiety symptoms in pregnant women, such as relationships between the pregnant woman and her in-laws, social support, sleep duration, personality traits, and history of abortion. Fourth, most of the respondents were from one province, and we did not obtain information from other regions, which would require further investigation. Finally, a previous study has demonstrated that COVID-19 is associated with depressive and anxiety symptoms [69], which were not included in the questionnaire in the study but might have affected the results.

## Conclusions

In summary, in the present study we demonstrated that the prevalence of depression and anxiety among Chinese pregnant women in the post-COVID-19 era was higher than expected and that sociodemographic contributors were among the risk factors for depressive and anxiety symptoms. More attention to the mental health of pregnant women is required from policymakers, along with greater awareness by families, to enhance early identification of mental health problems in pregnancy and develop mental health prevention programs for pregnant women, as important public health goals.

## Data Availability

The datasets used and/or analysed during the current study available from the corresponding author on reasonable request.
